# Changes in sleep architecture in German Armed Forces personnel with posttraumatic stress disorder compared with depressed and healthy control subjects

**DOI:** 10.1371/journal.pone.0215355

**Published:** 2019-04-17

**Authors:** Laura Haberland, Helge Höllmer, Holger Schulz, Kai Spiegelhalder, Robert Gorzka

**Affiliations:** 1 German Armed Forces Hospital Hamburg, Centre for Mental Health, Hamburg, Germany; 2 University Medical Centre Hamburg-Eppendorf, Department of Medical Psychology, Hamburg, Germany; 3 University Medical Centre Freiburg, Centre of Psychiatry and Psychotherapy, Freiburg, Germany; Stellenbosch University, SOUTH AFRICA

## Abstract

**Background:**

This study compares the sleep architecture of patients with posttraumatic stress disorder (PTSD) with that of both patients with depression and subjects with no mental disorder.

**Method:**

45 German armed forces personnel with PTSD, 72 German armed forces personnel with depression and 24 healthy control subjects underwent 24-hour polysomnography. The effects of group membership, medication and the statistical interaction of group and medication were analysed for the following variables: sleep onset latency, REM sleep latency, slow-wave sleep and REM sleep percentages.

**Results:**

Sleep onset latency was significantly prolonged in both the PTSD and the depression group. Moreover, psychotropic medication was associated with significantly prolonged REM sleep latency.

**Conclusion:**

The impact on sleep onset latency is of special clinical relevance in that according to preliminary studies, it is of major importance for subjective sleep quality. In contrast to the other parameters, an increase in sleep onset latency results in a subjective reduction in sleep quality which can lead to hyperarousal and increased preoccupation with sleep, which in turn may lead to dysfunctional sleep patterns.

## Introduction

Non-organic sleep disorders play an important part in mental illness. Numerous studies and systematic reviews have shown that pathological changes in sleep architecture play a major role as secondary symptoms in affective disorders [1], addiction [2] and schizophrenia [3]. It is especially after experiencing traumatic events that dyssomnias in the form of sleep-onset and sleep-maintenance insomnia as well as parasomnias such as nightmares are generally observed [4]. Studies of war veterans [5] and interviews of Holocaust survivors [6] suggest that such sleep disorders may persist for years after the traumatic event.

More detailed differentiation of sleep disorders is particularly required in terms of military medicine. As a result of shift duty, planned exercises and deployments abroad, military personnel are often faced with external changes to the sleep-wake-cycle, to which they must adjust. Interviews with military personnel have already shown the prevalence of sleep disorders to be higher among deployed military personnel than among the general population [7, 8, 9,10]. Moreover, the military profession and the reality of military operations involve an increased risk of developing mental illness and experiencing trauma. Increasing participation in active combat as part of a military mission has also increased the risk of developing post-traumatic disorders [11]. According to a recent study, the 12-month prevalence of PTSD in German soldiers deployed abroad is 2.9% [12], compared with 1.2% in non-deployed soldiers.

### Theoretical background

#### Sleep architecture

Polysomnography provides detailed information on sleep architecture. Each sleep phase shows periodically recurring EEG changes that define various sleep stages which recur several times during the night. The most commonly used classification today is based on that developed by Rechtschaffen and Kales in 1968. Individual sleep stages are classified based on EEG patterns in accordance with internationally recognised rules [13]. Sleep stages 1–4 as so-called NREM sleep are contrasted with REM sleep. Sleep stages 3 and 4 are summed up as slow-wave sleep by the American Academy of Sleep Medicine (AASM). REM sleep is characterised by a light sleep EEG pattern with the simultaneous occurrence of REM (**r**apid **e**ye **m**ovements) and (substantial) loss of skeletal muscle tone. REM sleep is similar to wakefulness in terms of basic EEG activity. However, unlike during awake EEG activity, people are difficult to rouse from REM sleep. The sequence of an NREM period with a subsequent REM phase is called a sleep cycle. During a night's sleep, we go through an average of about 4 to 6 cycles of about 90 minutes each [14]. Pathological changes in the latencies of sleep onset and REM sleep, deviating sleep phases in terms of duration and sequence as well as phases of nocturnal wakefulness (arousals) are among the sleep disorders detected by polysomnography.

#### PTSD and troubled sleep

According to the International Classification of Mental and Behavioural Disorders (ICD-10), posttraumatic stress disorder (F43.1) is defined as a reaction to trauma. In this context, trauma is described as "a stressful event or situation (of either brief or long duration) of an exceptionally threatening or catastrophic nature, which is likely to cause pervasive distress in almost anyone" [15]. The three characteristic main symptoms of this syndrome are intrusive stressful memories (e.g. nightmares, flashbacks), hyperarousal (hypervigilance, enhanced startle response, irritability), and trauma-associated avoidance behaviour [16]. Moreover, there is evidence that troubled sleep in PTSD patients is more than a secondary symptom and directly contributes to pathogenesis, especially as a result of arousal [17]. Other studies suggest that a premorbid sleep disorder may even be a risk factor for developing PTSD [18, 19].

Over the last 20 years, a number of studies in the field of sleep medicine have been carried out on patients with posttraumatic stress disorder [20]. Several recent surveys have highlighted both inconsistent results and methodological deficiencies. For one thing, many studies recruited only small samples of n<20, such as survivors of a natural disaster or disabled veterans [21], while larger samples are often inhomogeneous in terms of trauma latency (i.e. the time between the traumatic event and the development of symptoms) and age of traumatisation [8,9]. For another, many studies failed to record and statistically control important confounder such as patient age [22], comorbidities and medication, although sleep-inducing medication and comorbid mental illness are often concomitant factors, especially in trauma patients [23]. There are also important differences in the target variables so that the studies are not sufficiently comparable. The result is that abnormal movement during sleep and breathing difficulties are frequently conflated as sleep disorders, although they mostly have a different pathophysiology than, for instance, prolonged sleep onset latency. The lack of a general definition of the term of "sleep quality" is another challenge when it comes to comparing target variables, as polysomnography measures various parameters that are weighted differently in different study designs [18].

Meta analyses and surveys [4,19,24,25] have shown some polysomnographic studies of PTSD patients to yield inconsistent results. Some studies [9,26] provide evidence of disturbed sleep architecture (chronological sequence of sleep and wake stages), with shortened slow-wave sleep phases, increased sleep onset latency and REM sleep density (eye movement frequency during REM sleep). On the other hand, some studies of sleep efficiency and disturbances of the overall sleep architecture revealed no such disorders in PTSD patients [2]. Other studies have shown that PTSD patients not only go through prolonged and more frequent REM periods, but also more frequent brief wake phases (arousals) [21,27]. Particularly when it comes to REM sleep latency (occurrence of first REM sleep phase), results are inconsistent. Studies have revealed both an increase [26], a reduction or no significant change compared with control groups [5,21,28]. The parameter of REM sleep latency was chosen as the primary outcome in this study as it is believed to have a higher specificity with respect to post-traumatic disorders than sleep onset latency or slow-wave sleep percentage, which are examined in many other mental disorders, especially affective disorders. Moreover, many psychoactive drugs, especially TCAs and SSRIs, have a well-documented positive effect on REM latency [1]. A study by Klein et al. [29], however, only shows changes in sleep perception in PTSD patients but provides no polysomnographic evidence. Additionally, there is evidence that the severity of PTSD symptoms seems to correlate with sleep disorders measured with questionnaires [30]. There seems to be a discrepancy between subjective perception of a sleep disorder, i.e. as captured in standardised questionnaires normally used to diagnose sleep disorders (e.g. PSQI, LISD, ESS), and data obtained with polysomnography [29,30,31,32]. A recent study [33] came to the same conclusion and recommends combined analyses of objective and subjective data.

The ICD-10 diagnosis of nonorganic sleep disorder (F51) mainly refers to the latter as a "change in sleep perception", i.e. factors such as distress and excessive concern with the disorder are considered in addition to perceived poor sleep quality. Only the duration of sleep is included as an objectifiable parameter, albeit in a secondary role and without a precise definition (total sleep time, sleep period time).

### Control groups

The control groups in this study included not only healthy subjects but also soldiers with depressive disorders because they often experience sleep disorders such as delayed sleep onset, an increase in nighttime wake phases and early morning waking [34]. Moreover, there is evidence of an increased REM sleep percentage of sleep period time and reduced REM sleep latency with increased eye movement density, much like in PTSD patients [9,35]. Depressive disorders are the most common secondary diagnosis in PTSD patients and are not considered a criterion for exclusion in many studies despite their known impact on sleep architecture. This study, however, excluded depression as a comorbidity and integrated patients with depression as a separate control group in the analyses.

## Methods

### Sample

A consecutive sample of patients with an ICD-10 diagnosis of posttraumatic stress disorder (F43.1) or depression (F32.0, F32.1, F32.2, F33.0, F33.1, F33.2) were recruited from the pool of regular inpatients at the Mental Health Centre at German armed forces Hospital Hamburg from December 2014 until October 2017. Not only soldiers with deployment-related trauma were included in this study, but also those who developed PTSD with no (obvious) military context in accordance with ICD-10 diagnostic criteria. During their hospital stay, the patients were diagnosed by a psychiatrist based on the ICD-10 diagnostic criteria, moreover, there was a confirmation of the diagnosis by a psychologist. The following exclusion criteria were determined on the basis of ICD-10: psychotic disorder (F20-F29), manic episode, bipolar affective disorder (F30, F31), personality disorders (F60-F69), alcohol and/or drug addiction (F10-F19), neurological disease involving the central nervous system, sleep apnoea and other organic sleep disorders (G47.-) as well as nonorganic sleep disorders (F51.-) other than nightmares (F51.5). Furthermore, we excluded subjects who had been diagnosed with a comorbidity of the respective other group (i.e. PTSD or depression). Within the "PTSD" and "depression" groups, subjects taking psychoactive or other long-term medication known to influence sleep were recorded and analysed separately. These include antidepressants (TCAs = tricyclic antidepressants, SSRIs = selective serotonine reuptake inhibitors , SNRIs = selective serotonine and noradrenaline reuptake inhibitors, NDRIs = selective noradrenaline-dopamine reuptake inhibitors, NaSSA = noradrenergic specific serotonergic alpha2 receptor antagonist, MaSSA = melatonin specific serotonergic antagonist), one atypical neuroleptic (quetiapine), so-called Z-drugs (zolpidem, zopiclone) and, in one case, the anticonvulsant Pregabalin prescribed as a mood stabiliser. There is a well-documented effect of TCAs and SSRIs increasing the slow-wave sleep percentage of sleep period time at the expense of REM sleep and prolonging REM sleep latency [14,30]. For antidepressants such as TCA and Mirtazapine, the anticholinergic and above all the strong antihistaminergic effect contributes to this [1]. These groups of substances are also used in pharmacotherapy to treat PTSD patients. The so-called Z drugs have been demonstrated to result in sleep consolidation, i.e. improved sleep continuity and reduced nighttime waking, without a significant effect on slow-wave sleep [1].

The control group of healthy subjects, i.e. subjects without a relevant history of preexisting psychiatric or neurological disorders, consisted of personnel of the Mental Health Centre at German armed forces Hospital Hamburg and/or Helmut Schmidt German armed forces University in Hamburg. We were thus able to generate a sample that, apart from the control group (6 civilians), consisted solely of German armed forces military personnel, including 28 servicewomen and 113 servicemen. The average age was M_age_ = 30.3 years (SD_age_ = 8.1). In terms of level of education, 17.8% had completed higher education (n = 25), 19.1% had a general university entrance qualification (n = 27), 52.5% an intermediate secondary school-leaving certificate (n = 74), 9.9% a lower secondary school-leaving certificate (n = 14) and 0.7% no school-leaving qualification (n = 1).

During the date range we conducted 273 polysomnographies, 107 patients were excluded due to the exclusion criteria or due to the fact that the initial diagnosis was not confirmed during the hospital stay. 25 polysomnographies could not be analysed due to material artefacts, interruptions or stops of the recording.

At the outset, doctors informed the subjects of the scope and objective of the study (S1 Appendix). The study and data collection were approved by the ethics committee of the Medical Association of Hamburg, 9 December 2014.

### Polysomnography

The device used in this study for sleep monitoring was the Somnowatch TM plus. It includes a 6-channel electroencephalogram (F3, F4, C3, Cz, C4, P3, P4), an electromyogram to monitor muscle tone, an electrooculogram to track eye movements and actigraphy to monitor body positions and movements. Using this device, we can differentiate between REM sleep stages, sleep stage 1, sleep stage 2 and slow-wave sleep and determine their percentages of sleep duration and sequence throughout the sleep cycle. The following polysomnographic parameters were determined as target variables: sleep onset latency (reaching stage 2), REM sleep latency–with the parameters of sleep onset and REM sleep latency relating to the time from "lights out" (i.e. the time when the subject, according to the marker, lies down in bed to sleep) to reaching sleep stage 2 or REM sleep–slow-wave sleep percentage of sleep period time (sleep stages 3 and 4) and REM sleep percentage of sleep period time. Sleep period time (SPT) is the time from sleep onset to final awakening, including nighttime awakenings. Once the subjects had been informed and given their consent, they were briefed and had the device attached by a qualified sleep coach. Over the course of 24-hour recording, the device may be paused manually during certain activities that may cause motion artefacts (personal hygiene, etc.). The subjects should also list their activities throughout the recording period. Exact determination of sleep parameters also requires every subject to mark the time of "lights out" on the device. Visual control and any corrections of the analysis are performed by medical personnel experienced in EEG evaluation.

### Hypotheses

1. The group of PTSD patients significantly differs from the groups of depressed and healthy individuals in terms of the variables of sleep onset latency, REM sleep latency, REM sleep and slow-wave sleep.
2. The group of PTSD patients significantly differs in terms of the variables of sleep onset latency, REM sleep latency, REM sleep and slow-wave sleep from the group of depressed patients in the factor of medication under age control.
3. There is a interaction of group and medication on the variables of sleep onset latency, REM sleep latency, and percentages of REM sleep and slow-wave sleep.

The Sidak test was interpreted as a post-hoc test. The effect size was given by partial eta-squared (η^2^) with η^2^ > .01 being interpreted as a small effect, η^2^ > .06 as a medium effect and η^2^ > .14 as a large effect.

## Results

An analysis of covariance was performed to test hypothesis 1. The PTSD group was tested against the healthy and depressed groups. The age variable was used as a covariate. Table 1 shows significant differences of medium effect size between the three study groups in terms of sleep onset latency. Post-hoc analyses with Sidak adjustment yielded higher values for subjects of the PTSD group (M = 51.3, SD = 37.8) than for subjects of the healthy group (M = 20.1, SD = 15.9, p < .001). The same was observed in a comparison of the depressed group (M = 38.9, SD = 34.0) with the healthy group (p = .046). The PTSD group was not significantly different from the depressed group in terms of sleep onset latency (p = .113). With a small effect size, the REM sleep percentage of sleep period time was greater in the PTSD group than in the control group. The variance percentage explained by the age covariate was significant only for the variable of slow-wave sleep (p = .040).

**Table 1:** Differences between the three study groups in terms of sleep physiological variables (sleep onset latency, REM sleep latency, slow-wave sleep percentage of sleep period time, REM sleep percentage of sleep period time).

	PTSD Group n = 45		Depressed Group n = 72		Healthy Group n = 24				Age	
Variable	M	SD	M	SD	M	SD	F (2.14)	par. η^2^	F (1.14)	par. η^2^
Sleep onset latency (min)	51,3 a	37,8	38,9 b	34,0	20,1 a, b	15,9	7,33**	.097	0,62	.005
REM sleep latency (min)	92,4	51,0	93,6	59,7	83,0	41,1	0,38	.005	0,09	.001
REM sleep percentage (%)	22,2	9,3	19,5	7,5	20,8	5,7	1,73	.025	0,15	.001
Slow-wave sleep percentage (%)	20.2 ^adj^	9,2	20.2 ^adj^	8,7	21.9 ^adj^	6,4	0,38	.006	4,28*	.030

Mean values with the same superscript letters in one line significantly differ from each other in the post-hoc test (Sidak).

Since the variance percentage explained by the covariate was significant for the slow-wave sleep percentage variable, adjusted mean values are given

* p < .05

** p < .01.

To test hypotheses 2 and 3, a two-factor analysis of covariance with the factors diagnosis (PTSD vs. depression) and medication (sleep-inducing medication vs. no sleep-inducing medication) was performed and adjusted for age. The medication factor and the interaction between the diagnosis group and medication were tested.

Table 2 shows that for the medication factor, the variable REM sleep latency yielded a significant main effect F(1.117) = 5.089, p < .026, η^2^ = .043. The two groups of subjects who take medication showed higher values than the two groups who do not. The statistical interaction of group and medication was not significant for any of the four dependent variables.

**Table 2:** Effects of group membership, medication and interaction of group and medication on the sleep physiological variables (sleep onset latency, REM sleep latency, slow-wave sleep percentage of sleep period time, REM sleep percentage of sleep period time).

	PTSD Group n = 45 Medication				Depressed Group n = 72 Medication											
Variables	yes		no		yes		no									
	n = 19		n = 26		N = 26		n = 46		PTSD/Dep.		Med.		PTSD/Dep.*Med.		Age	
	M	SD	M	SD	M	SD	M	SD	F (1.117)	par. η^2^	F (1.117)	par. η^2^	F (1.117)	par. η^2^	F (1, 117)	par. η^2^
Sleep onset latency (min)	50,9	42,2	51,6	35,2	47,8	50,2	33,9	18,9	2,81	.024	1,01	.009	1,27	.011	1,13	.010
REM sleep latency (min)	99,0	54,3	87,7	49,0	117,2	74,8	80,3	44,9	0.19	.002	5,09*	.043	1,46	.013	1,13	.001
REM sleep percentage (%)	23,0	9,5	21,6	9,3	18,5	8,2	20,1	7,1	3,91*	.034	0,00	.000	0,75	.007	0,44	.004
Slow-wave sleep percentage (%)	18,7	10,1	20,5	8,6	18,7	8,6	21,3	8,6	0,01	.000	1,37	.012	0,01	.000	3,67	.032

Since the variance percentage explained by the covariate was not significant (all p >.058), mean values were not adjusted

* p < .05

## Discussion

The analysis results for the sleep onset latency variable show that this parameter is noticeably prolonged in subjects with PTSD as well as depression (differences with a small effect size, but not significant). While the literature suggests that this parameter is unspecific and pathologically prolonged in many mental disorders [3,34,35,36] it is nevertheless of special clinical relevance. Of all parameters analysed in this study, sleep onset latency is associated most closely with subjective sleep quality. Many studies show that an increase in slow-wave sleep (e.g. induced by medication) did not result in a major subjective increase in sleep quality [1], while a reduction in sleep onset latency and consolidation of sleep architecture, including a reduction in nighttime waking, did. According to Riemann et al. [17], prolonged sleep onset latency may lead to hyperarousal and increased preoccupation with sleep, which in turn may lead to dysfunctional sleep patterns. Questionnaires on sleep behaviour and subjective sleep quality have shown that PTSD patients in particular are strongly affected and that this may even correlate with the severity of PTSD symptoms [30]. This would suggest that, taking into account hyperarousal theory, disturbed sleep may not only be a posttraumatic symptom, but also a comorbidity that maintains the disorder or even a risk factor directly involved in its pathogenesis [18,19].

Because of numerous and partly inconsistent results of purely polysomnographic studies and the discrepancy between measured and subjectively perceived sleep quality shown in other studies, it is generally advisable to record both subjective and objective parameters [33].

As a primary outcome, a significant relationship between sleep-inducing medication and REM sleep latency has been demonstrated. REM sleep is thus much delayed in medicated patients. This is mainly due to the now well-documented effect of TCAs and SSRIs increasing the slow-wave sleep percentage of sleep period time at the expense of REM sleep and prolonging REM sleep latency [1]. These two groups of substances are also used in pharmacotherapy to treat PTSD patients. The effect on REM sleep suppression seems to be less pronounced (differences with small effect size, but not significant). PTSD patients are also frequently prescribed benzodiazepine agonists (so-called Z drugs) that have been demonstrated to result in sleep consolidation, i.e. improved sleep continuity and reduced nighttime waking, without a significant effect on slow-wave sleep.

The results concerning REM and slow-wave sleep percentages of sleep period time must be interpreted with caution. There are some minor effects and even significant differences of some 5%, but the time differences of 3 to 5 minutes reach practice-relevant levels.

No interaction of the factors PTSD / depression sleep-inducing medication could be demonstrated for all dependent variables (sleep onset latency, REM sleep latency, REM sleep percentage and slow-wave sleep percentage).

Furthermore, we performed all our analyses adjusted for age. Available data on changes in sleep patterns in old age show that physiological processes such as neurodegeneration of the suprachiasmatic nucleus, which serves as the neurological master clock, and the pineal gland as the site of melatonin production tend to result in reduced slow-wave and REM sleep periods and increased light sleep with prolonged sleep onset latencies and increased nighttime waking [22]. However, such degenerative processes as well as disorders that affect sleep, such as cataracts (through reduced light absorption via the eyes) or obstructive sleep apnoea syndrome, are only described for patients aged >60 years.

Methodological limitations: Owing to clinical constraints, EEG recording could not be performed during more than one night of the patients' hospital stay. The so-called 'first night effect' (FNE) describes a decrease in sleep time, slow-wave sleep and REM sleep percentages on account of changes in the natural sleeping environment (unfamiliar surroundings, sensors). As the FNE also refers to the gadget and equipment, the conditions are equal for patients and the healthy control group. Several studies have shown [37] that this effect is observed in both healthy individuals as well as those with sleep disorders, and that there may often be a 'second' or even 'third night effect', i.e. that differences in sleep parameters will occur even after two days of polysomnographic recording. We can assume that the effect was less pronounced in this study because most recordings took place after several days of inpatient stay.

Methodological strengths: One methodological strength of this study, and the one that is the most obvious in comparison with older studies, is its sample selection. The patient population is very homogenous in both trauma latency and age at traumatisation. Patients who were exposed to an event that led to PTSD according to ICD-10 diagnostic criteria in their childhood or adolescent years were excluded from this study. Furthermore, patients who take sleep-influencing medication were analysed separately. Although the literature is very clear on this issue, many studies ignore this factor or simply have psychotropic drugs, which are known to have a long half-life, cancelled or discontinued not until the day of the examination. A third advantage of the sample lies in its exclusion of secondary diagnoses and comorbidities that may influence sleep patterns. Depression in particular is a common secondary diagnosis of PTSD. In this study, it was analysed separately in a control group.

## Conclusion

In summary, the following conclusions can be drawn from this study:

As suggested by recent studies, a combined analysis is advisable because of the discrepancies between objective and subjective sleep disorders described above. It may even be wise to consider including this advice in the diagnostic criteria according to ICD-10. This suggestion is supported by the fact that a significantly prolonged sleep onset latency is perceived as an impairment. This is of practical relevance mainly in treatment (pharmacological and otherwise) that focuses on sleep onset. It seems to be recommended to deal with the changed sleep behaviour of the patients directly after the diagnosis and to take measures at an early stage, e.g. medication or even non medical sleep hygiene measurements to improve the process of falling asleep. If we take into account dysfunctional sleep caused by pathogenic hyperarousal (prolonged sleep onset latency), there is increasing evidence that disturbed sleep in PTSD patients is not just a secondary symptom, but a comorbidity.

## Supporting information

S1 Appendix Study protocol. (DOC)

S2 Appendix Information sheet. (DOC)

S3 Appendix Trend statement checklist. (DOC)
