# Supplementary material for: Changes in sleep architecture in German Armed Forces personnel with posttraumatic stress disorder compared with depressed and healthy control subjects
Source: PLoS One. 2019 Apr 17;14(4):e0215355. doi: 10.1371/journal.pone.0215355 (PMC6469790; doi:10.1371/journal.pone.0215355)
Supplement: S1 Appendix — (DOC) [file pone.0215355.s001.doc]

Bundeswehrkrankenhaus Hamburg

Abteilung VI b,
Zentrum für Seelische Gesundheit Hamburg

**Study protocol for the study : "Validation of Sleep Disorders in German Armed Forces soldiers with Post-Traumatic Stress Disorder by means of Polysomnography”**

1. **Presentation of the current state of knowledge**

Non-organic sleep disorders play a significant role in a variety of psychiatric disorders. Dysmosomes (1) and also parasomnias such as nightmares can be observed in particular in PTSD patients (2). In studies, there were indications of a disturbed sleep architecture with shortened deep sleep phases and a higher REM sleep density (3). However, meta-analyzes from the last few years have sometimes yielded contradictory results (9). A consistent finding seems to be an increased sleep latency, which may, however, also occur in a variety of other mental illnesses, such as depression.

Further studies have shown that PTSD patients not only had prolonged and more frequent REM periods, but also suffered from regularly shorter waking phases (arousals) (10,11). With regard to sleep efficiency and disorders of the overall sleep architecture, there are also studies which show no pathological changes in PTSD patients (12). The military environment plays a major role in this issue. Soldiers are often confronted with changes in the sleep-wake cycle due to shift work, exercise plans and assignments abroad, to which they have to adapt. Various studies have already shown that there is an increased prevalence of sleep disorders, especially among deployed soldiers compared to the normal population (4,5,6). On the other hand, the soldier's profession poses a high risk for the development of mental illness and traumatization due to the operational reality.

1. **Project purpose**

The aim of this dissertation is to differentiate between sleep disorders in soldiers with PTSD compared to healthy and depression-affected soldiers. The parameters of sleep latency, total duration of REM sleep and total duration of deep sleep should be investigated by means of polysomnography. The hypothesis is that in the group of PTSD patients the sleep latency is greater than in the “healthy and depressive” control group. In addition, it is to be examined whether in the group of PTSD patients the total duration of REM sleep is greater than in the control group healthy (and depressive) and if the total duration of deep sleep is smaller than in the control group healthy (and depressive)

In the context of this dissertation project, first of all, the focus will be placed on soldiers with PTSD based on operational conditions, and secondly a differentiation of sleep disorders by means of polysomnography. This consists of a long-term derivation of an EEG, EMG, EOG and actogrpahy. Here, electrophysiological parameters will be used to measure sleep continuity (the overall relationship between sleep and wakefulness), sleep latency (the time required for falling asleep), sleep efficiency (percentage of sleep and total time spent in bed) and sleep architecture (temporal and qualitative distribution of sleep stages). Also possible para- and dyssomnia can be detected and specified better. In addition to healthy volunteers, patients with depressive illness are also being examined as a control group, since sleep disturbances such as delayed falling asleep and early morning awakenings are regularly observed here. In addition, similar to PTSD patients, studies have shown an increased REM sleep density as well as a decreased REM sleep latency in polysomnography (7,8), so that the comparison group depressive seems apt here.

The proposed project aims to evaluate whether there are specific differences in sleep disorders in soldiers with post-traumatic stress disorder compared to those with depression and a healthy control group.
The specific parameters of sleep latency, total duration of deep sleep and total duration of REM sleep will be investigated by means of polysomnography.

The following theses were formulated:
1. In the group of PTSD patients, sleep latency is on average 20 minutes greater than in the control groups healthy (and depressive).
2. In the group of PTSD patients, the total duration of REM sleep is 20 minutes longer than in the control group healthy (and depressive).
3. In the group of PTSD patients, the total duration of deep sleep is 20 minutes shorter than in the control group healthy (and depressive).

1. **References**

Verwendete Literatur

(1) [van Liempt S](http://www.ncbi.nlm.nih.gov/pubmed?term=van Liempt S%5BAuthor%5D&cauthor=true&cauthor_uid=17853372)^1^, [Vermetten E](http://www.ncbi.nlm.nih.gov/pubmed?term=Vermetten E%5BAuthor%5D&cauthor=true&cauthor_uid=17853372), [de Groen JH](http://www.ncbi.nlm.nih.gov/pubmed?term=de Groen JH%5BAuthor%5D&cauthor=true&cauthor_uid=17853372), [Westenberg (2007)](http://www.ncbi.nlm.nih.gov/pubmed?term=Westenberg HG%5BAuthor%5D&cauthor=true&cauthor_uid=17853372)  Sleep disturbances in post-traumatic stress disorder. An overview of the literature

# (2) Krakow B, Hollifield M, Schrader R (2000). A controlled study of imagery rehearsal for chronic nightmares in sexual assault survivors with PTSD: a preliminary report. J Traum Stress 13:589-609.

# (3) Woodward SH, Bliwise DL, Friedman MJ, et al (1996). First night effects in post-traumatic stress disorder inpatients. Sleep 19:312-317.

(4) Luxton D, Greenburg D, Ryan J et al (2011) Prevalence and Impact of Short Sleep Duration in Redeployed OIF Soldiers. Sleep 34(9):1189-1195

(5) Peterson A L, Goodie M J L, Satterfield W A et al (2008) Sleep Disturbance during Military Deployment. Military Medicine 173:230-235

# (6) [Seelig AD](http://www.ncbi.nlm.nih.gov/pubmed?term=Seelig AD%5BAuthor%5D&cauthor=true&cauthor_uid=21120123)^1^, [Jacobson IG](http://www.ncbi.nlm.nih.gov/pubmed?term=Jacobson IG%5BAuthor%5D&cauthor=true&cauthor_uid=21120123), [Smith B](http://www.ncbi.nlm.nih.gov/pubmed?term=Smith B%5BAuthor%5D&cauthor=true&cauthor_uid=21120123), [Hooper TI](http://www.ncbi.nlm.nih.gov/pubmed?term=Hooper TI%5BAuthor%5D&cauthor=true&cauthor_uid=21120123), [Boyko EJ](http://www.ncbi.nlm.nih.gov/pubmed?term=Boyko EJ%5BAuthor%5D&cauthor=true&cauthor_uid=21120123), [Gackstetter GD](http://www.ncbi.nlm.nih.gov/pubmed?term=Gackstetter GD%5BAuthor%5D&cauthor=true&cauthor_uid=21120123), [Gehrman P](http://www.ncbi.nlm.nih.gov/pubmed?term=Gehrman P%5BAuthor%5D&cauthor=true&cauthor_uid=21120123), [Macera CA](http://www.ncbi.nlm.nih.gov/pubmed?term=Macera CA%5BAuthor%5D&cauthor=true&cauthor_uid=21120123), [Smith TC](http://www.ncbi.nlm.nih.gov/pubmed?term=Smith TC%5BAuthor%5D&cauthor=true&cauthor_uid=21120123); [Millennium Cohort Study Team](http://www.ncbi.nlm.nih.gov/pubmed?term=Millennium Cohort Study Team%5BCorporate Author%5D).(2010) Sleep patterns before, during, and after deployment to Iraq and Afghanistan.

# (7) William H. Spriggs: Sleep Technicians Pocket Guide: A Quick Reference Manual

# (8) S. Happe, B.W. Walther: Schlafmedizin in der Praxis

(9) Kobayashi I, Boarts JM, Delahanty DL. Polysomnographically Measured Sleep Abnormalities in PTSD: A meta-analytic review. Psychophysiologie 44 (2007)

(10) Ulmer C, Sutherland M, Edinger JD, Krystal A. REM sleep bout duration and frequency in those with PTSD. SLEEP, Volume 32, Abstact Supplement 1111 (2009)

(11) Bering R, Kuzmanovic B, Behmeburg C, Fischer G. Schlaf- und Traumastörungen – Psychotherapeutische und somatologische Behandlungsstrategien. Zeitschrift für Psychotraumatologie und Psychologische Medizin 4. JG Heft 1 (2006)

(12) Breslau N, Roth T, Burduvali E, Kapke A, Schultz L, Roehrs T. Sleep in Lifetime Posttraumatic Stress Disorder. Arch Gen Psyhciatry/Vol 61 (2004)

1. **Study planning and implementation**

Planned study period: 10 / 2014-02 / 2015

Polysomnography refers to the recording of various electrophysiological parameters (single-channel electroencephalogram, electromyogram, electrooculogram, and actography) for the recording of sleep stages as well as for the detection of eye movements and abnormal movement patterns as well as the position of the body during sleep. The polysomnography gives information inter alia on sleep continuity (total relationship between sleep and wakefulness), sleep latency (the time required for falling asleep, sleep efficiency (percentage of sleep and total time spent in bed), sleep architecture (temporal and qualitative distribution of different sleep stages).

With somnowatch ®, a single-channel EEG is recorded with EMG, EOG and nudeography, allowing complete differentiation of sleep stages into REM, 1,2,3 and 4, as well as their percentage in total sleep duration and the sequences throughout the sleep cycle. By determining the ratio of TST (total sleep time) to TIB (time in bed), the sleep efficiency parameter can be determined. By determining the sleep latency (from the time "lights out" according to protocol / mark replacement by the subject until the recording of the sleep stage 2). The aim of the research is to present a representative sample of patients with posttraumatic stress disorder (F43.1) and depressive illnesses F32, F33 as well as healthy volunteers. The sample will be recruited from the military personnel and psychiatric patients from the German Armed Forces Hospital in Hamburg or military personnel of the Helmut Schmidt University of the German Armed Forces in Hamburg. Patients are to be recruited from regular inpatient admissions. Based on a small drop-out rate of ≤5%, 25 patients should be recruited per test condition (total = 75).

Included diagnostic spectrum according to ICD-10:
Group 1: F43.1
Group 2: F32, F33
Group 3: healthy
The following exclusion criteria were established: psychotic disorder, bipolar disorder, alcohol and / or drug dependence, neurological disorders, sleep apnea syndrome, current drug treatment with sleep-inducing medication.

1. **Reasoned sample size**

The sample should include adult patients who have undergone in-patient diagnostics, assessment and / or treatment at the German Armed Forces Hospital Hamburg, Dept. VI B. Based on a small drop-out rate of ≤5%, 25 patients should be recruited per test condition (total n = 75).
Based on clinical experience values ​​for total REM sleep, sleep latency, and total deep sleep during the night, we find:
Average sleep latency: 12 minutes (SD 5.2)
Average total duration REM sleep per night: 90 minutes
Average total duration Deep sleep per night: 100 minutes
Assuming that the sleep latency is prolonged by 20 minutes (d = 1.6 according to Cohen), at alpha = 0.05 a sample size of n = 69 patients (per examination condition n = 23) is necessary. Based on a small drop-out rate of ≤5%, 25 patients should be recruited per test condition (total n = 75).
