# Supplementary material for: Changes in sleep architecture in German Armed Forces personnel with posttraumatic stress disorder compared with depressed and healthy control subjects
Source: PLoS One. 2019 Apr 17;14(4):e0215355. doi: 10.1371/journal.pone.0215355 (PMC6469790; doi:10.1371/journal.pone.0215355)
Supplement: S2 Appendix — (DOC) [file pone.0215355.s002.doc]

Bundeswehrkrankenhaus Hamburg

Abteilung VI b
Zentrum für Seelische Gesundheit Hamburg

Dipl-Psych. M Gorzka 040-6947-26440

label

**Education concernig the clinical trial Changes in sleep architecture in German Armed Forces personnel with posttraumatic stress disorder compared with depressed and healthy control subjects**

15.10.2014

Dear participant,

Being soldier and particularly deployment is highly connected with mental challenges and even mental disorders. Those often include sleep disorders. The participation is voluntary and can be recanted and cancelled anytime without any disadvantage. The data collection is part of the study and is conducted via “Polysomnography”. This means a electroencephalogram recording for 24 hours. You are going to be briefed and get the device attached by a qualified sleep coach. Pathological changes in the latencies of sleep onset and REM sleep, deviating sleep phases in terms of duration and sequence as well as phases of nocturnal wakefulness (arousals) are among the sleep disorders detected by polysomnography. The data and results are subject to medical confidentiality and data protection. The recording is going to be conducted with pseudonymisation. That means personal data and identification features as names or date of birth are going to be replaced with codes. Furthermore you have the possibility to get information about your data and the results. The study and data collection were approved by the ethics committee of the Medical Association of Hamburg. The federal state authority is able to get inspection in this clinical trial. After analysis, the pseudonymisation key is going to be deleted, and as a result, data are anonymized. In case of cancellation, data will be deleted as well.

Thank you for your support, Wirth best regards

🞎 I agree

🞎 I do not agree ______________________________

Datum, Unterschrift
