# Supplementary material for: Changes in sleep architecture in German Armed Forces personnel with posttraumatic stress disorder compared with depressed and healthy control subjects
Source: PLoS One. 2019 Apr 17;14(4):e0215355. doi: 10.1371/journal.pone.0215355 (PMC6469790; doi:10.1371/journal.pone.0215355)
Supplement: S3 Appendix — (DOC) [file pone.0215355.s003.DOC]

| ***Paper Section/Topic*** | ***Item No.*** | | ***Descriptor*** |  |
| --- | --- | --- | --- | --- |
| ***TITLE and ABSTRACT*** | | | |  |
| *Title and Abstract* | *1* | | - *Information on how units were allocated to interventions* | *Changes in sleep architecture in German Armed Forces personnel with posttraumatic stress disorder compared with depressed and healthy control subjects* |
|  |  | | - *Structured abstract recommended* | *Background: This study compares the sleep architecture of patients with posttraumatic stress disorder (PTSD) with that of both patients with depression and subjects with no mental disorder.*  *Method: 45 Bundeswehr personnel with PTSD, 72 Bundeswehr personnel with depression and 24 healthy control subjects underwent 24-hour polysomnography. The effects of group membership, medication and group x medication interaction were analysed for the following variables: sleep onset latency, REM sleep latency, slow-wave sleep and REM sleep percentages.*  *Results: Sleep onset latency was significantly prolonged in both the PTSD and the depression group. Moreover, psychotropic medication was associated with significantly prolonged REM sleep latency and the REM sleep period was longer in the PTSD group than in the depression group.*  *Conclusion: The impact on sleep onset latency is of special clinical relevance in that according to preliminary studies, it is of major importance for subjective sleep quality. Longer sleep onset latency in the PTSD group may not only be a trauma sequela, but may also be involved in maintaining the disorder.* |
|  |  | | - *Information on target population or study sample* | *A consecutive sample of patients with an ICD-10 diagnosis of posttraumatic stress disorder (F43.1) or depression (F32, F33) were recruited from the pool of regular inpatients at the Mental Health Centre at Bundeswehr Hospital Hamburg.*  *The control group of healthy subjects, i.e. subjects without a relevant history of preexisting psychiatric or neurological disorders, consisted of personnel of the Mental Health Centre at Bundeswehr Hospital Hamburg and/or Helmut Schmidt Bundeswehr University in Hamburg.* |
| ***INTRODUCTION*** | | | |  |
| *Background* | *2* | - *Scientific background and explanation of rationale* | | *Over the last years, a number of studies in the field of sleep medicine have been carried out on patients with posttraumatic stress disorder. Several recent surveys have highlighted both inconsistent results and methodological deficiencies. Additionally, there seems to be a discrepancy between subjective perception of a sleep disorder, i.e. as captured in standardised questionnaires normally used to diagnose sleep disorders and data obtained with polysomnography. The lack of a general definition of the term of "sleep quality" is another challenge when it comes to comparing target variables, as polysomnography measures various parameters that are weighted differently in different study designs* |
|  |  | - *Theories used in designing behavioral interventions* | |  |
| ***METHODS*** | | | |  |
| *Participants* | *3* | | - *Eligibility criteria for participants, including criteria at different levels in recruitment/sampling plan (e.g., cities, clinics, subjects)* | *A consecutive sample of patients were recruited from the pool of regular inpatients at the Mental Health Centre at Bundeswehr Hospital Hamburg. The study and data collection were approved by the ethics committee of the Medical Association of Hamburg (9 December 2014).* |
|  |  | | - *Method of recruitment (e.g., referral, self-selection), including the sampling method if a systematic sampling plan was implemented* | *The following exclusion criteria were determined on the basis of ICD-10: psychotic disorder (F20-F29), manic episode, bipolar affective disorder (F30, F31), personality disorders (F60-F69), alcohol and/or drug addiction (F10-F19), neurological disease involving the central nervous system, sleep apnoea and other organic sleep disorders (G47.-) as well as nonorganic sleep disorders (F51.-) other than nightmares (F51.5). Furthermore, we excluded subjects who had been diagnosed with a comorbidity of the respective other group (i.e. PTSD or depression). Within the "PTSD" and "depression" groups, subjects taking psychoactive or other long-term medication known to influence sleep were recorded and analysed separately.* |
|  |  | | - *Recruitment setting* |  |
|  |  | | - *Settings and locations where the data were collected* | *Mental Health Centre at Bundeswehr Hospital Hamburg and/or Helmut Schmidt Bundeswehr University in Hamburg* |
| *Interventions* | *4* | | - *Details of the interventions intended for each study condition and how and when they were actually administered, specifically including:* |  |
|  |  | | - - *Content: what was given?* | *The device used in this study for sleep monitoring was the Somnowatch TM plus ®. It includes a 6-channel electroencephalogram (F3, F4, C3, Cz, C4, P3, P4), an electromyogram to monitor muscle tone, an electrooculogram to track eye movements and actigraphy to monitor body positions and movements. Using this device, we can differentiate between REM sleep stages, sleep stage 1, sleep stage 2 and slow-wave sleep and determine their percentages of sleep duration and sequence throughout the sleep cycle. Once the subjects had been informed and given their consent, they were briefed and had the device attached by a qualified sleep coach. Visual control and any corrections of the analysis are performed by medical personnel experienced in EEG evaluation.* |
|  |  | | - - *Delivery method: how was the content given?* |  |
|  |  | | - - *Unit of delivery: how were subjects grouped during delivery?* |  |
|  |  | | - - *Deliverer: who delivered the intervention?* |  |
|  |  | | - - *Setting: where was the intervention delivered?* |  |
|  |  | | - - *Exposure quantity and duration: how many sessions or episodes or events were intended to be delivered? How long were they intended to last?* |  |
|  |  | | - - *Time span: how long was it intended to take to deliver the intervention to each unit?* |  |
|  |  | | - - *Activities to increase compliance or adherence (e.g., incentives)* |  |
| *Objectives* | *5* | | - *Specific objectives and hypotheses* | 1. *The group of PTSD patients significantly differs from the groups of depressed and healthy individuals in terms of the variables of sleep onset latency, REM sleep latency, REM sleep and slow-wave sleep.* 2. *The group of PTSD patients significantly differs in terms of the variables of sleep onset latency, REM sleep latency, REM sleep and slow-wave sleep from the group of depressed patients in the factor of medication under age control.* 3. *There is a group x medication interaction effect on the variables of sleep onset latency, REM sleep latency, and percentages of REM sleep and slow-wave sleep.* |
| *Outcomes* | *6* | | - *Clearly defined primary and secondary outcome measures* | *The parameter of REM sleep latency was chosen as the primary outcome. Further variables: sleep onset latency, REM sleep latency, slow-wave sleep and REM sleep percentages.* |
|  |  | | - *Methods used to collect data and any methods used to enhance the quality of measurements* |  |
|  |  | | - *Information on validated instruments such as psychometric and biometric properties* | *An analysis of covariance was performed to test hypothesis 1. The PTSD group was tested against the healthy and depressed groups. The age variable was used as a covariate. To test hypotheses 2 and 3, a two-factor analysis of covariance with the factors diagnosis (PTSD vs. depression) and medication (sleep-inducing medication vs. no sleep-inducing medication) was performed and adjusted for age. The medication factor and the interaction between the diagnosis group and medication were tested.* |
| *Sample size* | *7* | | - *How sample size was determined and, when applicable, explanation of any interim analyses and stopping rules* | *The sample should include adult patients who have undergone in-patient diagnostics, assessment and / or treatment at the German Armed Forces Hospital Hamburg, Dept. VI B. Based on a small drop-out rate of ≤5%, 25 patients should be recruited per test condition (total n = 75).* |
| *Assignment method* | *8* | | - *Unit of assignment (the unit being assigned to study condition, e.g., individual, group, community)* |  |
|  |  | | - *Method used to assign units to study conditions, including details of any restriction (e.g., blocking, stratification, minimization)* |  |
|  |  | | - *Inclusion of aspects employed to help minimize potential bias induced due to non-randomization (e.g., matching)* | *Random counterchecking by a second evaluator of the polysomnographical data.* |
| *Blinding (masking)* | *9* | | - *Whether or not participants, those administering the interventions, and those assessing the outcomes were blinded to study condition assignment; if so, statement regarding how the blinding was accomplished and how it was assessed* |  |
| *Unit of Analysis* | *10* | | - *Description of the smallest unit that is being analyzed to assess intervention effects (e.g., individual, group, or community)* |  |
|  |  | | - *If the unit of analysis differs from the unit of assignment, the analytical method used to account for this (e.g., adjusting the standard error estimates by the design effect or using multilevel analysis)* |  |
| *Statistical methods* | *11* | | - *Statistical methods used to compare study groups for primary methods outcome(s), including complex methods for correlated data* | *An analysis of covariance was performed to test hypothesis 1. The PTSD group was tested against the healthy and depressed groups. The age variable was used as a covariate. To test hypotheses 2 and 3, a two-factor analysis of covariance with the factors diagnosis (PTSD vs. depression) and medication (sleep-inducing medication vs. no sleep-inducing medication) was performed and adjusted for age. The medication factor and the interaction between the diagnosis group and medication were tested. Post-hoc analyses with Sidak adjustment was performed.* |
|  |  |  | - *Statistical methods used for additional analyses, such as subgroup analyses and adjusted analysis* |  |
|  |  |  | - *Methods for imputing missing data, if used* |  |
|  |  |  | - *Statistical software or programs used* |  |
| ***RESULTS*** | | | |  |
| *Participant flow* | *12* | | - *Flow of participants through each stage of the study: enrollment, assignment, allocation and intervention exposure, follow-up, analysis (a diagram is strongly recommended)* |  |
|  |  | | - - *Enrollment: the numbers of participants screened for eligibility, found to be eligible or not eligible, declined to be enrolled, and enrolled in the study* | *We were thus able to generate a sample that, apart from the control group (6 civilians), consisted solely of Bundeswehr military personnel, including 28 servicewomen and 113 servicemen. The average age was M_age_ = 30.3 years (SD_age_ = 8.1). In terms of level of education, 17.8% had completed higher education (n = 25), 19.1% had a general university entrance qualification (n = 27), 52.5% an intermediate secondary school-leaving certificate (n = 74), 9.9% a lower secondary school-leaving certificate (n = 14) and 0.7% no school-leaving qualification (n= 1).* |
|  |  | | - - *Assignment: the numbers of participants assigned to a study condition* |  |
|  |  | | - - *Allocation and intervention exposure: the number of participants assigned to each study condition and the number of participants who received each intervention* |  |
|  |  | | - - *Follow-up: the number of participants who completed the follow-up or did not complete the follow-up (i.e., lost to follow-up), by study condition* |  |
|  |  | | - - *Analysis: the number of participants included in or excluded from the main analysis, by study condition* |  |
|  |  | | - *Description of protocol deviations from study as planned, along with reasons* |  |
| *Recruitment* | *13* | | - *Dates defining the periods of recruitment and follow-up* |  |
| *Baseline data* | *14* | | - *Baseline demographic and clinical characteristics of participants in each study condition* | *The average age was M_age_ = 30.3 years (SD_age_ = 8.1). In terms of level of education, 17.8% had completed higher education (n = 25), 19.1% had a general university entrance qualification (n = 27), 52.5% an intermediate secondary school-leaving certificate (n = 74), 9.9% a lower secondary school-leaving certificate (n = 14) and 0.7% no school-leaving qualification (n= 1).* |
|  |  | | - *Baseline characteristics for each study condition relevant to specific disease prevention research* |  |
|  |  | | - *Baseline comparisons of those lost to follow-up and those retained, overall and by study condition* |  |
|  |  | | - *Comparison between study population at baseline and target population of interest* |  |
| *Baseline equivalence* | *15* | | - *Data on study group equivalence at baseline and statistical methods used to control for baseline differences* |  |
| *Numbers analyzed* | *16* | | - *Number of participants (denominator) included in each analysis for each study condition, particularly when the denominators change for different outcomes; statement of the results in absolute numbers when feasible* | *PTSD group n=42, Depression group n= 72, Healthy group n=24* |
|  |  | | - *Indication of whether the analysis strategy was “intention to treat” or, if not, description of how non-compliers were treated in the analyses* |  |
| *Outcomes and estimation* | *17* | | - *For each primary and secondary outcome, a summary of results for each estimation study condition, and the estimated effect size and a confidence interval to indicate the precision* | *For the medication factor, the variable* ***REM sleep latency*** *yielded a significant main effect F(1.117) = 5.089, p < .026, η² = .043. The two groups of subjects who take medication showed higher values than the two groups who do not.*  *There are significant differences of medium effect size between the three study groups in terms of* ***sleep onset latency****. Post-hoc analyses with Sidak adjustment yielded higher values for subjects of the PTSD group (M = 51.3, SD = 37.8) than for subjects of the healthy group (M = 20.1, SD = 15.9, p < .001). The same was observed in a comparison of the depressed group (M = 38.9, SD = 34.0) with the healthy group (p = .046). The PTSD group was not significantly different from the depressed group in terms of sleep onset latency (p = .113). With a small effect size, the* ***REM sleep percentage*** *of sleep period time was greater in the PTSD group than in the control group. The variance percentage explained by the age covariate was significant only for the variable of slow-wave sleep (p = .040).* |
|  |  | | - *Inclusion of null and negative findings* | *To test hypotheses 2 and 3, a two-factor analysis of covariance with the factors diagnosis (PTSD vs. depression) and medication (sleep-inducing medication vs. no sleep-inducing medication) was performed and adjusted for age. The medication factor and the interaction between the diagnosis group and medication were tested.*  *The group x medication interaction factor was not significant for any of the four dependent variables.* |
|  |  | | - *Inclusion of results from testing pre-specified causal pathways through which the intervention was intended to operate, if any* |  |
| *Ancillary analyses* | *18* | | - *Summary of other analyses performed, including subgroup or restricted analyses, indicating which are pre-specified or exploratory* |  |
| *Adverse events* | *19* | | - *Summary of all important adverse events or unintended effects in each study condition (including summary measures, effect size estimates, and confidence intervals)* |  |
| ***DISCUSSION*** | | | |  |
| *Interpretation* | *20* | | - *Interpretation of the results, taking into account study hypotheses, sources of potential bias, imprecision of measures, multiplicative analyses, and other limitations or weaknesses of the study* | *The analysis results for the* ***sleep onset latency*** *variable show that this parameter is noticeably prolonged in subjects with PTSD as well as depression (differences with a small effect size, but not significant). While the literature suggests that this parameter is unspecific and pathologically prolonged in many mental disorders, it is nevertheless of special clinical relevance. Of all parameters analysed in this study, sleep onset latency is associated most closely with subjective sleep quality. As a primary outcome, a significant relationship between* ***sleep-inducing medication and REM sleep latency*** *has been demonstrated. REM sleep is thus much delayed in medicated patients, particularly those in the depressed group. This is mainly due to the now well-documented effect of TCAs and SSRIs increasing the slow-wave sleep percentage of sleep period time at the expense of REM sleep and prolonging REM sleep latency. These two groups of substances are also used in pharmacotherapy to treat PTSD patients. PTSD patients are also frequently prescribed benzodiazepine agonists (so-called Z drugs) that have been demonstrated to result in sleep consolidation, i.e. improved sleep continuity and reduced nighttime waking, without a significant effect on slow-wave sleep*  *Because of numerous and partly inconsistent results of purely polysomnographic studies and the discrepancy between measured and subjectively perceived sleep quality shown in other studies, it is generally advisable to record both subjective and objective parameters..*  *The results concerning* ***REM and slow-wave sleep percentages*** *of sleep period time must be interpreted with caution. There are some minor effects and even significant differences of some 5%, but the time differences of 3 to 5 minutes reach practice-relevant levels.*  *No interaction of the factors PTSD / depression sleep-inducing medication could be demonstrated for all dependent variables (sleep onset latency, REM sleep latency, REM sleep percentage and slow-wave sleep percentage).*  *Furthermore, we performed all our analyses adjusted for* ***age****. Available data on changes in sleep patterns in old age show that physiological processes such as neurodegeneration of the suprachiasmatic nucleus, which serves as the neurological master clock, and the pineal gland as the site of melatonin production tend to result in reduced slow-wave and REM sleep periods and increased light sleep with prolonged sleep onset latencies and increased nighttime waking. However, such degenerative processes as well as disorders that affect sleep, such as cataracts (through reduced light absorption via the eyes) or obstructive sleep apnoea syndrome, are only described for patients aged >60 years.* |
|  |  | | - *Discussion of results taking into account the mechanism by which the intervention was intended to work (causal pathways) or alternative mechanisms or explanations* |  |
|  |  | | - *Discussion of the success of and barriers to implementing the intervention, fidelity of implementation* | *Methodological limitations: Owing to clinical constraints, EEG recording could not be performed during more than one night of the patients' hospital stay. The so-called 'first night effect' (FNE) describes a decrease in sleep time, slow-wave sleep and REM sleep percentages on account of changes in the natural sleeping environment (unfamiliar surroundings, sensors). However, several studies have shown that this effect is observed in both healthy individuals as well as those with sleep disorders, and that there may often be a 'second' or even 'third night effect', i.e. that differences in sleep parameters will occur even after two days of polysomnographic recording. Furthermore, we can assume that the effect was less pronounced in this study because most recordings took place after several days of inpatient stay.*  *Methodological strengths: One methodological strength of this study, and the one that is the most obvious in comparison with older studies, is its sample selection. The patient population is very homogenous in both trauma latency and age at traumatisation. Patients who were exposed to an event that led to PTSD according to ICD-10 diagnostic criteria in their childhood or adolescent years were excluded from this study. Furthermore, patients who take sleep-influencing medication were analysed separately* |
|  |  | | - *Discussion of research, programmatic, or policy implications* |  |
| *Generalizability* | *21* | | - *Generalizability (external validity) of the trial findings, taking into account the study population, the characteristics of the intervention, length of follow-up, incentives, compliance rates, specific sites/settings involved in the study, and other contextual issues* |  |
| *Overall evidence* | *22* | | - *General interpretation of the results in the context of current evidence and current theory* | *In summary, the following conclusions can be drawn from this study:*  *As suggested by recent studies, a combined analysis is advisable because of the discrepancies between objective and subjective sleep disorders described above. It may even be wise to consider including this advice in the diagnostic criteria according to ICD-10. This suggestion is supported by the fact that a significantly prolonged sleep onset latency is perceived as an impairment. This is of practical relevance mainly in treatment (pharmacological and otherwise) that focuses on sleep onset. If we take into account dysfunctional sleep caused by pathogenic hyperarousal (prolonged sleep onset latency), there is increasing evidence that disturbed sleep in PTSD patients is not just a secondary symptom, but a comorbidity that maintains the disorder or even a risk factor.* |
